# Truncated *PPM1D* impairs stem cell response to genotoxic stress and promotes growth of APC-deficient tumors in the mouse colon

**DOI:** 10.1038/s41419-019-2057-4

**Published:** 2019-10-28

**Authors:** Monika Burocziova, Kamila Burdova, Andra S. Martinikova, Petr Kasparek, Petra Kleiblova, Stine A. Danielsen, Marianna Borecka, Gabriela Jenikova, Lucie Janečková, Jozef Pavel, Petra Zemankova, Michaela Schneiderova, Lucie Schwarzova, Ivana Ticha, Xiao-Feng Sun, Katerina Jiraskova, Vaclav Liska, Ludmila Vodickova, Pavel Vodicka, Radislav Sedlacek, Zdenek Kleibl, Ragnhild A. Lothe, Vladimír Korinek, Libor Macurek

**Affiliations:** 1 Cancer Cell Biology, Institute of Molecular Genetics of the Czech Academy of Sciences, Prague, Czech Republic; 2 Czech Centre for Phenogenomics, Institute of Molecular Genetics of the Czech Academy of Sciences, Prague, Czech Republic; 3 Institute of Biochemistry and Experimental Oncology, First Faculty of Medicine, Charles University, Prague, Czech Republic; 4 Department Molecular Oncology, Institute for Cancer Research, & K.G. Jebsen Colorectal Cancer Research Centre, Oslo University Hospital, Oslo, Norway; 5 Cell and Developmental Biology, Institute of Molecular Genetics of the Czech Academy of Sciences, Prague, Czech Republic; 6 Department of Abdominal, Thoracic Surgery and Traumatology, First Faculty of Medicine, Charles University and General Faculty Hospital in Prague, Prague, Czech Republic; 7 Department of Endocrinology and Metabolism, First Faculty of Medicine, Charles University and General Faculty Hospital in Prague, Prague, Czech Republic; 8 Department of Oncology and Department of Clinical and Experimental Medicine, Linköping University, Linköping, Sweden; 9 Institute of Pathology, First Faculty of Medicine, Charles University and General University Hospital in Prague, Prague, Czech Republic; 10 Molecular Biology of Cancer, Institute of Experimental Medicine, ASCR, Prague, Czech Republic; 11 Biomedical Centre, Faculty of Medicine in Pilsen, Charles University, Prague, Czech Republic; 12 Institute of Biology and Medical Genetics, First Medical Faculty, Charles University, Prague, Czech Republic

**Keywords:** Cancer models, Checkpoints

## Abstract

Protein phosphatase magnesium-dependent 1 delta (PPM1D) terminates cell response to genotoxic stress by negatively regulating the tumor suppressor p53 and other targets at chromatin. Mutations in the exon 6 of the *PPM1D* result in production of a highly stable, C-terminally truncated PPM1D. These gain-of-function *PPM1D* mutations are present in various human cancers but their role in tumorigenesis remains unresolved. Here we show that truncated PPM1D impairs activation of the cell cycle checkpoints in human non-transformed RPE cells and allows proliferation in the presence of DNA damage. Next, we developed a mouse model by introducing a truncating mutation in the *PPM1D* locus and tested contribution of the oncogenic *PPM1D*^*T*^ allele to colon tumorigenesis. We found that p53 pathway was suppressed in colon stem cells harboring *PPM1D*^*T*^ resulting in proliferation advantage under genotoxic stress condition. In addition, truncated PPM1D promoted tumor growth in the colon in *Apc*^min^ mice and diminished survival. Moreover, tumor organoids derived from colon of the *Apc*^min^*Ppm1d*^*T/+*^ mice were less sensitive to 5-fluorouracil when compared to *Apc*^*min*^*Ppm1d*^*+/+*^and the sensitivity to 5-fluorouracil was restored by inhibition of PPM1D. Finally, we screened colorectal cancer patients and identified recurrent somatic *PPM1D* mutations in a fraction of colon adenocarcinomas that are p53 proficient and show defects in mismatch DNA repair. In summary, we provide the first in vivo evidence that truncated PPM1D can promote tumor growth and modulate sensitivity to chemotherapy.

## Introduction

Genome instability is one of the hallmarks of cancer^1^. In the presence of DNA damage, healthy cells protect their genome integrity by arresting progression through the cell cycle and by activation of specialized DNA repair pathways^2,3^. Double-strand DNA breaks (such as those generated by ionizing radiation, IR) and replication stress (for example induced by oncogenes) activate ataxia telangiectasia-mutated kinase (ATM) and ataxia telangiectasia and Rad3-related kinase (ATR), respectively. Subsequently, phosphorylation and ubiquitination-dependent recruitment of various proteins [such as TP53-binding protein 1 (53BP1) and Breast cancer type-1 susceptibility protein (BRCA1)] to the flanking chromatin enables DNA repair by non-homologous end joining or homologous recombination. In addition, ATM/ATR jointly activate tumor suppressor protein p53 triggering expression of its target genes that control cell fate decisions, including duration of the cell cycle arrest, senescence, or programmed cell death^4^. Functional DNA damage response (DDR) prevents proliferation of cells in the presence of DNA damage and thus represents an intrinsic barrier preventing oncogenic transformation^5,6^. Conversely, molecular defects in DDR as well as inactivating mutations in the *TP53* gene (coding for p53 protein) lead to genome instability, promote tumor development and can affect the therapeutic response^2,4,7^.

Protein phosphatase magnesium-dependent 1 delta (PPM1D; called also Wip1) is a negative regulator of p53 that allows timely termination of the G2 checkpoint^8–10^. Loss of *Ppm1d* protected mice from development of MMTV-Erb2-driven mammary tumors, Eµ-myc-induced B-cell lymphomas and *Apc*^*min*^-driven intestinal adenocarcinoma^11–14^. Inactivation of *Ppm1d* increased p53-, checkpoint kinase 2 (CHK2)-, and growth arrest and DNA damage gene 45 alpha (GADD45A)-dependent apoptosis of the intestinal stem cells (ISCs) and prevented their transformation into tumor-initiating stem cells^12,13^. Conversely, amplification of the *PPM1D* locus (17q23.2) leading to overexpression of PPM1D phosphatase was observed in about 10% of human breast cancers and several other cancer types^15–17^. Typically, overexpression of PPM1D occurs in p53-proficient tumors suggesting that suppression of the p53 pathway is the major role of the phosphatase during oncogenesis^15^. In addition to amplification of the *PPM1D* locus, nonsense mutations in exon 6 of *PPM1D* leading to production of the C-terminally truncated protein were recently reported in human cancers^18–21^. Since the C-terminal truncation does not affect enzymatic activity of PPM1D nor its subcellular distribution, truncated PPM1D protein can access its physiological substrates at chromatin^18^. In particular, heterozygous truncating mutations in the *PPM1D* are present in several p53-proficient cancer cell lines (including U2OS and HCT116 cells) and disable activation of the G1 checkpoint^18^. Gain-of-function phenotype of the truncated PPM1D is caused by abnormally prolonged protein half-life due to the loss of a degradation motif located in the last 65 amino acids of PPM1D^18,22^. Besides somatic mutations, age-related truncating mutations in *PPM1D* occur in a fraction of hematopoietic stem cells (HSCs) leading to clonal hematopoiesis^22,23^. The importance of these mutations is highlighted in mutation carriers receiving chemotherapy, because HSCs carrying the truncated *PPM1D* show better survival and potentially may allow development of secondary cancers including acute myeloid leukemia (AML) and myelodysplastic syndrome^23,24^.

Most of the supporting evidence for oncogenic properties of PPM1D comes from cell-based assays or from the knock-out mouse model, however, contribution of the truncated PPM1D to tumor growth has not been addressed in vivo so far. Here we generated a mouse model mimicking the truncating mutation in *PPM1D* identified in human cancers. Subsequently, we studied the impact of truncated Ppm1d on cell response to DNA damage, as well as its ability to potentiate colon carcinoma growth in vivo. We show that truncated Ppm1d can suppress p53-mediated response in ISCs. As a result, ISCs carrying the mutated *Ppm1d*^*T*^ allele survive in the presence of genotoxic stress better than the wild-type ISCs. In addition, *Ppm1d*^*T/+*^ mice showed accelerated growth of *Apc*^*min*^-driven adenocarcinoma in the colon. Tissue organoids derived from tumors expressing truncated PPM1D were resistant to 5-fluorouracil (5-FU), whereas they responded well to combined treatment with 5-FU and a small-molecule inhibitor of PPM1D. Finally, we identified recurrent somatic truncating *PPM1D* mutations in a fraction of human colon adenocarcinomas that were associated with defects in mismatch DNA repair pathway (MMR), while retaining wild type (wt) p53. In summary, we provide the first in vivo evidence that truncation of PPM1D contributes to tumorigenesis and may affect response of tumor cells to chemotherapy.

## Materials and methods

### Ethical approval

All animal models and experiments of this study were ethically reviewed and approved by the Institute of Molecular Genetics (c.j. 1/2016). All tumor samples were provided from subjects that gave their written informed consent approved by the local ethical committees and the research complies with the Declaration of Helsinki. The project was approved by the Regional Committee for Medical and Health Research Ethics, South Eastern Norway (REC number 1.2005.1629; 2010/1805) and the Norwegian Data Inspectorate.

### Patient samples

A total of 947 primary CRC from three series were analyzed for gene mutations and MSI. Fresh-frozen tumor specimens were consecutively collected from patients (*n* = 364) treated for primary CRC (stage I–IV) at Oslo University Hospital in the period December 2005–June 2011. Czech cohort consisted of 243 non-selected sporadic CRC patients. Tumor tissue and adjacent non-malignant tissue were resected during primary surgery and collected to the dry ice. Swedish cohort included 340 primary CRC tissue and paired distant normal mucosa from patients diagnosed at the University Hospital in Linköping and Vrinnevi Hospital in Norrköping. Representative tumor tissues, evaluated by pathologist, were stored for subsequent analyses at dry ice.

### Gene mutation analyses

Variants affecting *PPM1D* exon 6 were identified as described previously^18^. Briefly, DNA from non-cancer mucosa and colorectal tumor tissues were isolated by a routine procedure and was PCR amplified in two overlapping amplicons covering *PPM1D* exon 6 and directly sequenced. Paired non-cancer and tumor samples with identified *PPM1D* variants were subjected for analysis by next generation sequencing (NGS) using CZECANCA panel targeting 219 cancer-predisposition and candidate genes and bioinformatic analysis was performed as described^25^. All de novo indels identified in the tumor samples were visually inspected in IGV software. Recurrent *TP53*, *KRAS*, and *BRAF-V600E* mutations were identified by a routine pathological assessment in tumor samples and retrieved from patients’ records when available or analyzed from tumor DNA as described previously^26–29^. Microsatellite instability (MSI) status of the tumors was determined using the consensus markers provided by the National Cancer Institute (Bethesda marker panel), as previously described^27^.

### Cell lines

hTERT-RPE1 (RPE) cells were obtained from ATCC, immortalized human colon cells from Applied Biological Materials (Cat. no. T0570) and HCT116 cells from Dr. Medema (NKI, Amsterdam). For Clustered Regularly Interspaced Short Palindromic Repeats (CRISPR)/Cas9-mediated editing, RPE cells or human colon cells were transfected with pSpCas9-(BB)-2A-Puro plasmid (Addgene ID48139) carrying targeting sequences GAAGGCATTGCTACGAACCAGGG (CR1) or ATAGCTCGAGAGAATGTCCAAGG (CR2) located in the exon 6 of *PPM1D* and single cell clones were expanded^30^. Alternatively, RPE cells were transfected with a combination of pZGB-3L-EGFP and pZGB-3R-mCherry plasmids containing coding sequence for FokI nuclease and transcription activator-like effector nuclease (TALEN) repeats targeting TGAAGAAAATTGCGCTA and TCAAAGAATC-ATGTATC regions in exon6 of human *PPM1D* (ZgenBio Biotech, Taiwan). Cells positive for mCherry and EGFP were sorted by Influx instrument (BD Biosciences) and single cell clones were expanded. Correct editing in the targeted region was confirmed by sequencing of genomic DNA and the stabilization of PPM1D protein by immunoblotting. RPE-TP53-KO cells with knocked-out *TP53* were generated as described^31^. Relative cell proliferation after various treatments was determined by resazurin assay as described previously^31^.

### Cell cycle checkpoint

Cells were pulsed with 5-ethynyl-2′-deoxyuridine (EdU, Jena Bioscience) and subsequently exposed to ionizing radiation generated by Precision X-RAD 225XL (dose 3, 6, or 10 Gy) and were grown for further 20 h in the presence of nocodazole (250 ng/mL). Cells were stained with anti-pSer10-histone H3 (mitotic marker, Santa Cruz) and 4′,6-diamidino-2-phenylindole (DAPI as a marker of DNA content) and EdU was labeled with AlexaFluor 647 using Click-iT reaction (Thermo Scientific). Cells were analyzed by flow cytometry using LSRII (BD Biosciences) and FlowJo software (FlowJo). EdU-positive cells were assayed for progression through the G2 phase to mitosis (4n DNA content, pH3+). EdU-negative cells with 2*n* content were used for quantification of cells arrested in G1 checkpoint.

### Antibodies and reagents

Following antibodies were used: WIP1 (clone D4F7) Rabbit mAb (Cell Signaling, #11901) for detection of mouse PPM1D, WIP1 antibody (clone F-10, Santa Cruz sc-376257) for detection of human PPM1D, Importin-β (Santa Cruz, sc-137016), p21 for immunoblotting (Santa Cruz, sc-397), p21 (BD pharmingen, #556431) for IHC, p53 (clone D01, Santa Cruz, sc-126), phospho-S15-p53 (Cell Signaling, #9284), RPA2 (Abcam), phospho-S824-KAP1 (Genetex, GTX63711), KAP1 (Genetex, GTX62973), 14-3-3 (Santa Cruz, sc-133233), γH2AX (Millipore, 05-636 and Cell Signaling, #9718), TFIIH (Santa Cruz, sc-293), and Ki-67 (Genetex, GTX16667).

### Animals

Mice were maintained in the animal facility of the Institute of Molecular Genetics on a standard diet and 12 h light–dark cycle with free access to food and water. Ppm1d mutant mice were generated by TALEN-mediated genome editing^32^. The following TALEN-repeat domain sequences were used to target exon 6 of *Ppm1d* gene: left TALEN: NI NI NG NN HD HD NG NG HD NG HD NI NN NI NN NI NI NN, right TALEN: NG HD HD HD NG HD NG NI NN HD NG NI NG HD NG HD NI NN HD. RNA precursors of TALENs were microinjected into male nucleoli of zygotes isolated from C57BL/6NCrl mice as described^33^. These zygotes were subsequently implanted into pseudopregnant females. Tail biopsies from newborn pups were tested for the presence of TALEN-mediated frameshift mutations using Sanger sequencing. Founder mouse carrying a c.1324_1339del16 frameshift-inducing mutation (resulting in p.F442Lfs*3) within exon 6 of *Ppm1d* gene was further bred to establish Ppm1d mutant line (referred to as *Ppm1d*^*T/+*^). *Ppm1d*^*T/+*^ mice were further crossed with previously described strains *Apc*^*Min/J*^, *Lgr5*^*tm1(cre/ERT2)Cle*^ (Lgr5-EGFP-IRES-CreERT2 mice) and *Trp53*^*tm1Tyj*^ (p53 knock-out mice)^34–36^. Animals were sacrificed at indicated times or when tumor burden was apparent or when moribund. Where indicated, mice or cells were irradiated with Precision X-RAD 225XL (dose 3 or 4 Gy as indicated; Cu filter 0.5 mm). For immunoblotting, mouse tissues were lyzed in 250 mM Tris–HCl (pH 6.8), 40% glycerol and 8% SDS buffer.

### Genotyping

DNA was isolated from the tails by incubating overnight at 56 °C in 200 μL of a lysis solution containing [50 mM Tris pH 8.0, 100 mM EDTA, and 0.5% SDS] supplemented with proteinase K (100 μg/mL), precipitated by 0.5 M NaCl and isopropanol and resuspended in water.

Genotypes were determined by PCR. PCR fragment was digested by AvaII restriction enzyme, to distinguish between wt *Ppm1d*^*+/+*^ (cut) or *Ppm1d*^*T/+*^ (uncut) alleles. Following PCR primers were used: *Ppm1d:* CTAAGGACCATATACCTGCCCTTGTTCGCAG, and GATAGTATTT-GTTGAATTGGTTGGAATGAGGCC; *Apc*^*min*^: common primer GCCATCCCTTCACGTTAG and TTCCACTTTGGCATAAGGC specific for wt allele or TTCTGAGAAAGACAGAAGTTA specific for mutant allele; *Tp53*: common primer GAAACAGGCTAACCTAACCTACCACGC and ATCCCGAGTATCTGGAAGACAGGC specific for wt allele or TTTGAATGGAAGGATTGGAGCTACGG specific for mutant allele; *Lgr5*: common primer CTGCTCTCTGCTCCCAGTCT and ATACCCCATCCCTTTTGAGC specific for wt allele or GAACTTCAGGGTCAGCTTGC specific for mutant allele.

### Immunohistochemistry

The colon was harvested and placed in a 4% formaldehyde diluted in PBS overnight and then transferred into 70% ethanol. Paraffin-embedded colon sections were cut 4 μm thick and deparaffinized, cleared, and rehydrated in graded ethanol concentrations. Antigen retrieval was performed in a steam bath in Na–citrate buffer (10 mM, pH 6.0) for 30 min. Sections were pretreated with peroxidase blocking buffer (3% H_2_O_2_ in methanol) for 20 min at room temperature (RT). Blocking of non-specific binding was performed using 5% BSA for 1 h at RT. All antibodies were incubated overnight at 4 °C. Tissue sections were stained with p21 (BD Pharmingen #556431), Ki-67 (GeneTex #GTX16667), γH2A.X (Cell Signaling #9718). Secondary anti-rabbit-Alexa-488/594 or Biotin-XX Goat anti-Rabbit IgG biotin-conjugated (Thermo Fisher Scientific) were applied for 1 h at RT. TUNEL assay was performed using the In Situ Cell Death Detection kit (Invitrogen, #10618). To examine the morphological changes in the colon, standard haematoxylin and eosin (H&E) staining protocol was followed.

### Isolation of colon crypts

Isolation of colon crypts and the dissociation of cells for flow cytometry analysis were performed as previously described^37^. In brief, colon crypts were isolated from 8 weeks old mice. Luminal contents were flushed out with cold PBS, tissue was split open longitudinally and incubated in 5 mM EDTA (pH 8) in PBS at 4 °C for 90 min. To isolate single cells from colon crypts, the pellet was further incubated with Dispase (Thermo Scientific; stock solution 100 mg/mL diluted 1:300 in serum-free DMEM) using rigorous shaking on rotating platform (800 RPM, 3 × 5 min, 37 °C). Large pieces of colon were then allowed to settle by gravity for 1 min, leaving the isolated crypts in suspension. These were collected and retained. Supernatant was diluted in DMEM containing 10% FBS (Gibco), and passed through a 70-μm strainer (Corning) to obtain a single-cell suspension. The step was repeated three times until the supernatant appeared clear. The sample was centrifuged for 5 min at 300 × *g* and cells were resuspended in PBS.

### Flow cytometry and cell sorting

Sorting of ISCs and differentiated cells from small intestine or colon was performed as we described previously^38,39^. The single-cell suspension of colon crypts from Lgr5-EGFP-IRES-CreERT2 mice was stained with phycoerythrin-conjugated anti-CD24 antibody (Thermo Fisher Scientific, #12-0242-81) for 30 min at 4 °C. Sorting was performed using an Influx cell sorter (BD Biosciences) and cells were gated by forward scatter (FSC), side scatter (SSC), and negative staining for Hoechst 33258. The crypt base colon stem cells were sorted according to the cell size, CD24 stem cell marker and GFP signal (CD24^+^/GFP^+^) and compared to differentiated cells (CD24^−^/GFP^−^). Alternatively, single-cell suspension from small intestinal crypts was stained with antibody against Ulex europaeus agglutinin (UEA), marker of Paneth cells, and ISCs were sorted according to GFP^+^/UEA^−^ signal.

### Real-time quantitative reverse transcriptase PCR (qRT-PCR)

Colon mucosa was homogenized using TissueLyser LT (Qiagen) and RNA was extracted with the RNAeasy Mini Kit (Qiagen) according to the manufacturer’s instructions and cDNA was synthesized using the 0.5 μg RNA, random hexamer, and RevertAid H Minus Reverse Transcriptase (Thermo Scientific). Alternatively, RNA was isolated from ISCs or differentiated cells (5 × 10^3^ cells) sorted by flow cytometry using RNeasy Micro Kit (Qiagen) and cDNA was generated using MAXIMA Reverse Transcriptase (Thermo Scientific). RT-qPCR was performed using LightCycler 480 SYBR Green I Master mix, Light Cycler LC480 (Roche, Basel, Switzerland) and following cycle conditions: initial denaturation 95 °C for 7 min, followed by 45 cycles of denaturation 95 °C for 15 s, annealing 60 °C for 15 s and extension 72 °C for 15 s. Melting curve analysis was used to confirm the specificity of amplification, and cycle threshold (Ct) values were determined using LigtCycler480 software. All qRT-PCR experiments were performed in triplicates and data are presented as the ratio of the tested mRNA to *glyceraldehyde-3-phosphate dehydrogenase* (GAPDH) mRNA. Following primers were used for qRT-PCR: *Ppm1d*: AGCCAGGAGACCTGTGTGAT and GGCATTACTGCGAACAAGGG; *CDKN1A*: TGAGGAGGAGCATGAATGGAGACA and AACAGGTCGGACATCACCAGGATT; *PUMA*: CCTGGAGGGTCATGTACAATCT and TGCTACATGGTGCAGAAAAAGT; *GAPDH*: TGGCAAAGTGGAGATTGTTGCC and AAGATGGTGATGGGCTTCCCG; *LGR5*: CAAGCCATGACCTTGGCCCTG and TTTCCCAGGGAGTG-GATTCTATT.

### Organoid culture

Organoid cultures were established from freshly isolated colon tumors from 16 weeks old mice as described previously^37,40^. The organoids were embedded in Matrigel (BD Biosciences) and cultured with Advanced DMEM/F12 supplemented with penicillin/streptomycin, 10 mM HEPES, 2 mM GlutaMAX, N-2 supplement, B27 supplement (all Thermo Scientific), N-acetylcysteine (1 mM; Sigma-Aldrich), mRspo1 (500 ng/mL; Peprotech), mNoggin (100 ng/mL; Peprotech), and primocin (100 μg/mL, Invivogen). The following niche factors were added to the basal culture medium depending on the niche requirements of CRC organoid lines: gastrin I (10 nM; Sigma Aldrich #G9145), mouse recombinant EGF (50 ng/mL; Invitrogen Biosource #PMG8043), TGFβ type I receptor inhibitor (500 nM; Sigma Aldrich #A83-01) and p38 inhibitor (3 µM; Sigma Aldrich #SB202190). Tumor organoid proliferation was measured using resazurin proliferation assay. Organoids were seeded and embedded in Matrigel in 96-well plates. Following day, organoids were treated with 5-FU (20 µM; Sigma) and with DMSO or GSK2830371 (Medchemexpress). After 3 days, a mixture of resazurin (30 μg/mL) and complete organoid medium was added to each well, incubated for 2 h and fluorescence signal (Ex/Em = 540/590 nm) was measured by Envision plate reader (PerkinElmer). Samples were measured in hexaplicates and three independent experiments were performed.

### Tumor size determination

The small intestine and colon of 16-week-old mice of indicated genotypes were resected longitudinally, washed with cold PBS and examined for polyp number count and tumor size. Length, width, and height of the tumor were measured by vernier caliper and tumor volume was calculated using formula (L*W*H)/2. The investigator was blinded to the group allocation during the experiment.

### Statistical analysis

Each experiment was repeated at least three times and statistical significance was evaluated using Graph Pad Prism 5.04 software. Unless stated otherwise, two-sided unpaired *t*-test was used and *p* values < 0.05 are considered statistically significant. Kaplan–Meier survival plot was evaluated using log rank test. Error bars indicate standard deviation.

## Results

### Truncation of PPM1D impairs the checkpoint and p53 response in human cells

**Fig. 1:**
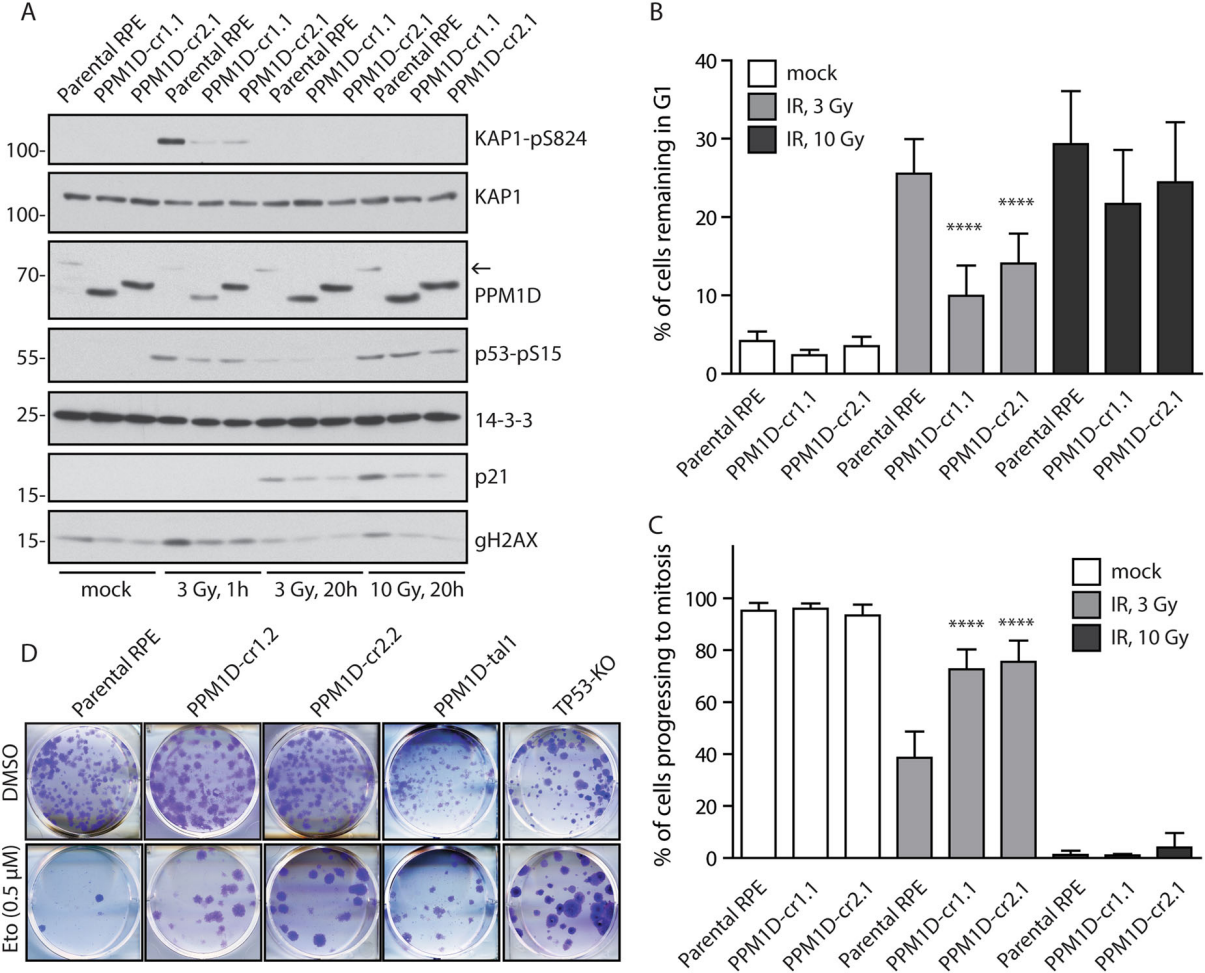
Truncation of PPM1D impairs the checkpoint and p53 response in human cells. **a** Parental RPE and RPE-PPM1D-cr1.1 and RPE-PPM1D-cr2.1 cells were exposed to 3 or 10 Gy of IR, collected at indicated times and analyzed by immunoblotting. Arrow indicates position of the full length PPM1D. **b** Parental RPE and RPE-PPM1D-cr1.1 and RPE-PPM1D-cr2.1 cells were pulse labeled by EdU and 20 h after exposure to 3 or 10 Gy of IR were analyzed by flow cytometry. Plotted are EdU negative cells with 2n DNA content that correspond to cells arrested in G1. Bars indicate SD (*n* = 3). **c** Parental RPE and RPE-PPM1D-cr1.1 and RPE-PPM1D-cr2.1 cells were pulse labeled by EdU and 20 h after exposure to 3 or 10 Gy of IR were analyzed by flow cytometry. Plotted are pS10-histone H3-positive cells from EdU-positive cells corresponding to cells that progressed from S phase to mitosis. Bars indicate SD (*n* = 3). **d** Parental RPE and RPE-PPM1D-cr1.2, RPE-PPM1D-cr2.2 and RPE-PPM1D-tal1 cells were seeded in six-well plate and treated with etoposide for 24 h. Subsequently fresh media was added and colony formation was assayed after 14 days

We have previously shown that heterozygous truncating mutations in exon 6 of the *PPM1D* impair activation of the p53 pathway in several human cancer cell lines, including U2OS and HCT116 cells^18^. Since cancer cells are genetically instable, we tested whether PPM1D truncation would cause similar defects in non-transformed cells. To this end, we introduced truncating mutations in exon 6 of the *PPM1D* gene in hTERT-RPE1 (RPE) cells using CRISPR/Cas9 technology and evaluated cell response to IR (Fig. 1, Supplementary Fig. S1a, b). Truncated PPM1D was expressed at considerably higher level than the full-length protein which is in agreement with the proposed role of the C-terminal degron motif in PPM1D turn over^18,22^. As expected, cells carrying the stabilizing PPM1D mutation showed lower level of KAP1-pS824, p53-pS15, and γH2AX phosphorylation, three established PPM1D targets (Fig. 1a)^9,41^. In addition, these cells failed to activate p53 and did not arrest in the G1 checkpoint after exposure to IR (Fig. 1b). Cells with truncated PPM1D also entered prematurely to mitosis after exposure to a low dose of IR which was consistent with the established role of p53 in maintenance of the G2 checkpoint^42^ (Fig. 1c). However, high dose of IR allowed full activation of the G2 checkpoint suggesting that the negative impact of the PPM1D activity on the p53 pathway can be compensated by high activity of upstream ATM/ATR kinases. We observed similar effects of the truncated PPM1D on DDR in independent RPE clones where genome editing was performed by TALEN technology (Supplementary Fig. S1c–e), and in immortalized human colon cells (Supplementary Fig. S1f, g), suggesting that mechanisms controlling PPM1D stability and its function in negative regulation of the p53 pathway do not depend on specific genetic or cellular background. Next, we performed colony formation assays to study the long-term effects of PPM1D truncation on cell proliferation under genotoxic stress. Whereas proliferation of the wt RPE cells was efficiently suppressed by a DNA damage-inducing drug etoposide, RPE cells producing truncated PPM1D formed comparable amount of colonies as RPE cells with inactivated p53 (Fig. 1d).

### Truncation of PPM1D impairs response to genotoxic stress in the mouse colon

**Fig. 2:**
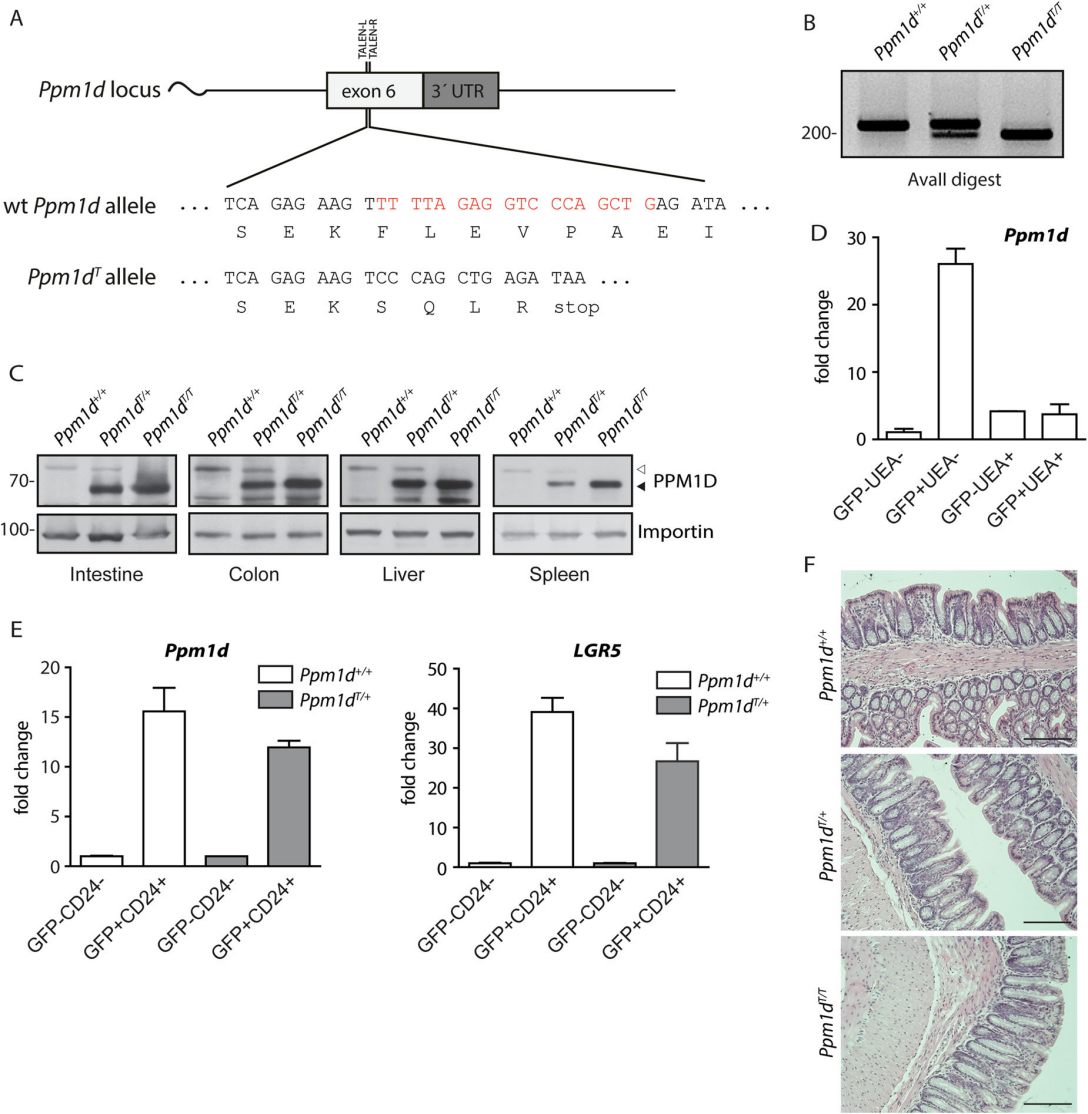
Wild-type and truncated *PPM1D* are highly expressed in ISCs in mouse. **a** Schematic representation of TALEN-mediated editing of the *Ppm1d* locus. DNA and protein sequences of the wild type *Ppm1d*^*+*^ and *Ppm1d*^*T*^ alleles are shown. Bases lost by editing are indicated in red. **b** Genotyping of F2 generation. Exon 6 of *Ppm1d* was PCR amplified from *Ppm1d*^*+/+*^, *Ppm1d*^*T/+*^, and *Ppm1d*^*T/T*^ mice and subjected to AvaII digestion. **c** Protein levels of wt and truncated PPM1D in intestine, colon, liver, and spleen were analyzed by western blotting. Empty and full arrowhead indicate positions of wt and truncated PPM1D, respectively. Importin was used as a loading control. **d** Expression of *Ppm1d* was determined by RT-qPCR in ISCs (GFP^+^UEA^−^), Paneth cells (GFP^−^UEA^+^) and other differentiated cells isolated from small intestine of Lgr5-EGFP-IRES-CreERT2 mice and was normalized to the *GAPDH* expression levels. **e** Expression of *Ppm1d* was determined by RT-qPCR in colon ISCs (GFP^+^CD24^+^) and differentiated colon cells (GFP^−^CD24^−^) isolated from Lgr5-EGFP-IRES-CreERT2-*Ppm1d*^*+/+*^ or Lgr5-EGFP-IRES-CreERT2-*Ppm1d*^*T/+*^ and was normalized to *GAPDH* (*n* = 3). **f** Representative H&E-stained sections of the colon from 8 weeks old wt *Ppm1d*^*+/+*^, *Ppm1d*^*T/+*^, and *Ppm1d*^*T/T*^ mice

**Fig. 3:**
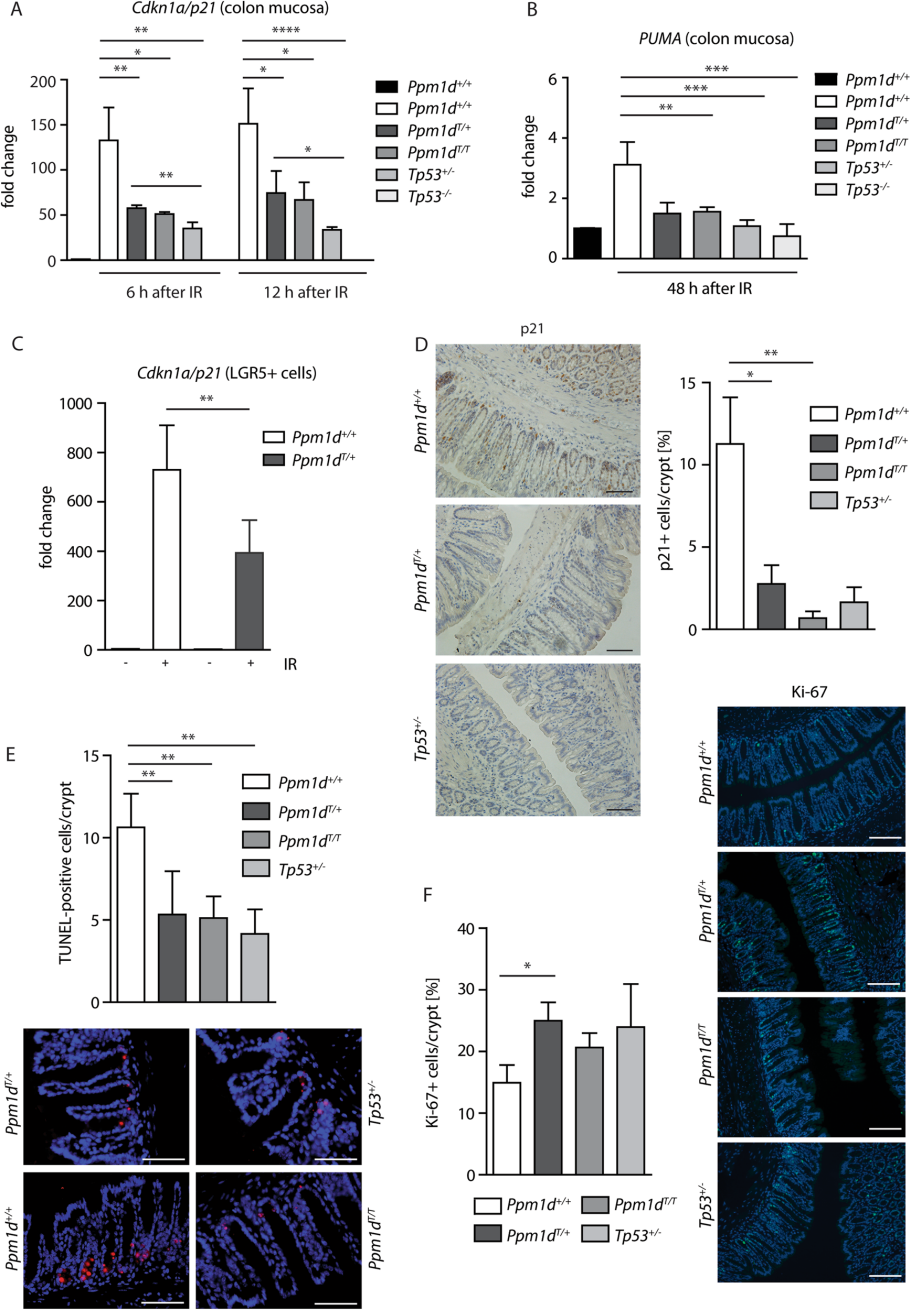
Truncation of *PPM1D* impairs response to ionizing radiation in the mouse colon. **a** Expression of *Cdkn1a* in colon mucosa 6 and 12 h after exposure of mice of indicated genotypes to 3 Gy of IR was measured by RT-qPCR and normalized to *GAPDH* (*n* ≥ 3). b) Expression of *PUMA* in the colon mucosa 48 h after exposure to 3 Gy of IR was measured by RT-qPCR and normalized to *GAPDH* (*n* ≥ 3). **c** LGR5-EGFP-positive ICS cells were sorted from colon of wt *Ppm1d*^*+/+*^, and *Ppm1d*^*T/+*^ mice exposed or not to 3 Gy of IR and expression of *Cdkn1a* was measured by RT-qPCR and normalized to *GAPDH* (*n* = 3). **d** Levels of p21 were determined in the mouse colon 48 h after exposure to 3 Gy of IR by immuno-histochemistry. The percentage of p21-positive cells per crypt was scored (*n* ≥ 4). At least 90 crypts were counted for each group. Representative images are shown. Scale bar 25 µm. **e** Apoptotic cells were detected in colon epithelia of mice of indicated genotypes 48 h after exposure to IR (3 Gy) by TUNEL apoptosis assay (*n* = 5). At least 90 crypts were counted for each group. Representative images are shown. Scale bar 50 µm. **f** Levels of a proliferation marker Ki-67 were determined in the mouse colon 48 h after exposure to 3 Gy of IR by immuno-histochemistry (*n* ≥ 4). At least 90 crypts were counted for each group. Representative images are shown. Scale bar 50 µm

Next, we aimed to determine the impact of truncated PPM1D in the tissue context of and to evaluate its potential role in cancer growth. To this end, we employed TALEN technology and generated a mouse strain producing the C-terminally truncated PPM1D (Fig. 2a, b). Similarly to human cells, heterozygote *Ppm1d*^*T/+*^ mice showed higher level of truncated Ppm1d protein compared to the full-length Ppm1d in all tested tissues including small intestine, colon, liver, and spleen (Fig. 2c). Expression of *Ppm1d* was previously detected by in situ RNA hybridization at the bottom of the crypts in small intestine^12^. This compartment is composed of terminally differentiated Paneth cells and self-renewing ISCs; the latter cells specifically express leucine-rich repeat-containing G protein-coupled receptor 5 (LGR5)^34,43,44^. We used Lgr5-EGFP-IRES-CreERT2 mice carrying either wt or truncated *Ppm1d* to isolate different subpopulations of cells from intestinal epithelium and analyzed *PPM1D* expression. Using real-time qRT-PCR, we confirmed that *Ppm1d* mRNA was highly expressed in the small intestinal ISCs (EGFP^+^/UEA^−^) but not in Paneth cells (EGFP^−^/UEA^+^) (Fig. 2d). Similarly, *Ppm1d* was highly expressed in colon ISCs double positive for Lgr5-EGFP and a surface antigen CD24 (Fig. 2e). Next, we performed histological analysis of the wt and *Ppm1d*^*T/+*^ mice and found that truncation of PPM1D did not impair the general organization of the small intestinal and colon epithelium (Fig. 2f). To study the role of PPM1D in response to genotoxic stress, we exposed mice carrying the *Ppm1d*^*T*^ allele to IR and analyzed expression of candidate genes in the isolated colon mucosa. As expected, expression of *Cdkn1a* (coding for the cell cycle inhibitor p21) was highly induced in wt animals after exposure to a low dose of IR (Fig. 3a). In contrast, expression of *Cdkn1a* was significantly decreased in *Ppm1d*^*T/+*^ mice exposed to IR (Fig. 3a). Interestingly, the suppression of *Cdkn1a* in *Ppm1d*^*T/+*^ was similar to the level observed in *Tp53*^*+/−*^ mice suggesting that truncated PPM1D can partially inhibit the p53 pathway in the mouse colon (Fig. 3a). In addition, truncated PPM1D-impaired expression of *PUMA*, an established p53 target and the main pro-apoptotic gene in ISCs (Fig. 3b)^45,46^. Furthermore, expression analysis performed in LGR5^+^ cells isolated from mice exposed or not to IR revealed lower expression of *Cdkn1a* and two pro-apoptotic genes *Trp53inp1* and *PHLDA3* in *Ppm1d*^*T/+*^ confirming that truncated Ppm1d impairs the p53-dependent response of colon ISCs to genotoxic stress (Fig. 3c, Supplementary Fig. S2)^47,48^. In agreement with the expression analysis, we observed lower amounts of p21-positive cells in the colon crypts of *Ppm1d*^*T/+*^ mice using immunohistochemistry (Fig. 3d). Similarly, we detected lower amounts of apoptotic cells in the colon crypts of *Ppm1d*^*T/+*^ mice after exposure to IR compared to wt mice (Fig. 3e). Conversely, we found higher levels of the proliferation marker Ki-67 in the *Ppm1d*^*T/+*^ colon epithelium when compared to wt mice (Fig. 3f), which was consistent with the role of truncated PPM1D in blocking p53-dependent suppression of the cell proliferation and survival.

### Truncation of PPM1D promotes growth of colon tumors in Apc^min^ mice

**Fig. 4:**
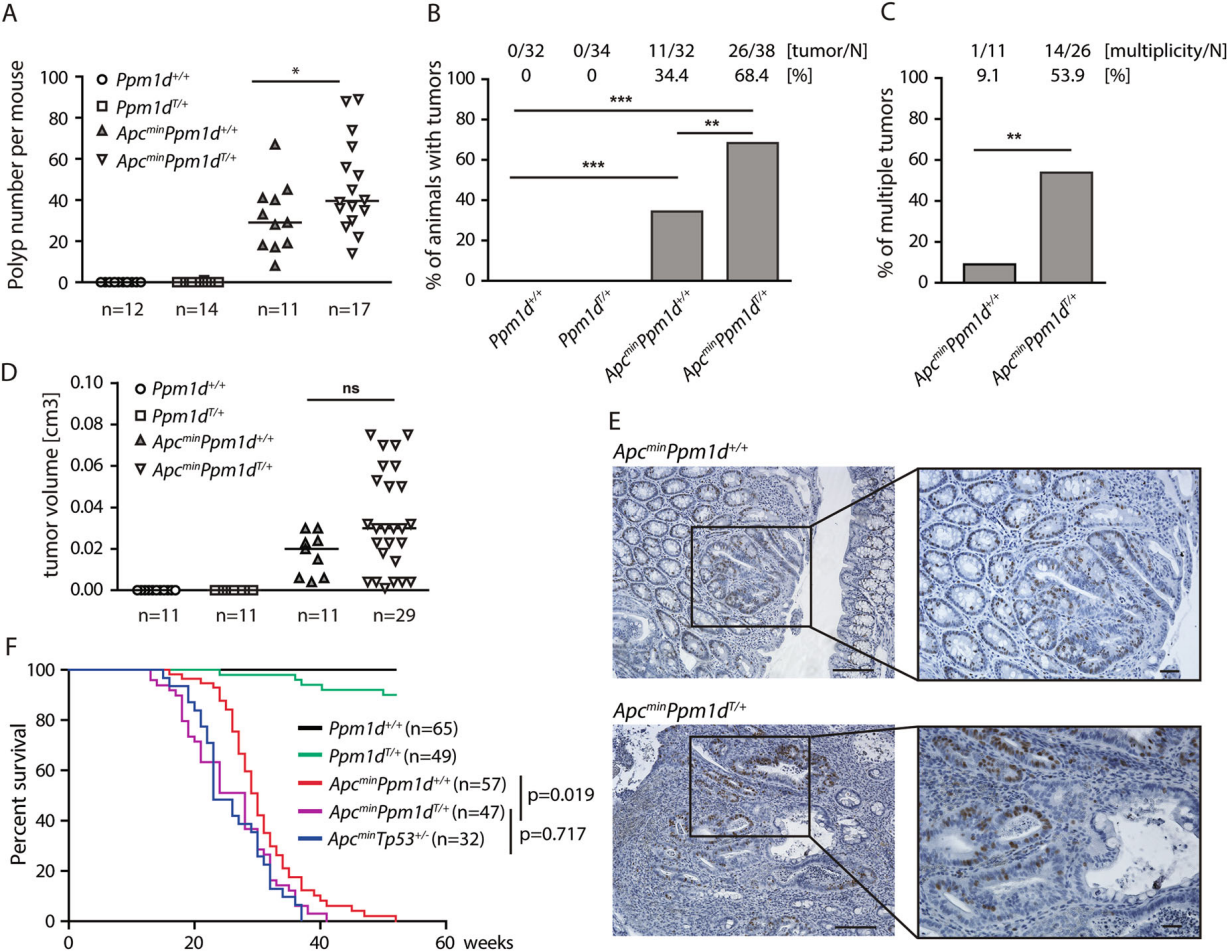
Truncation of *PPM1D* promotes growth of APC-induced colon tumors. **a** Wild-type *Ppm1d*^*+/+*^*, Ppm1d*^*T/+*^*, Apc*^*min*^, and *Apc*^*min*^*Ppm1d*^*T/+*^ mice were sacrificed at 16 weeks and the number of intestinal polyps was scored (*p* = 0.044). Numbers indicate numbers of animals of each genotype. **b** Wild-type *Ppm1d*^*+/+*^*, Ppm1d*^*T/+*^*, Apc*^*min*^, and *Apc*^*min*^*Ppm1d*^*T/+*^ mice were sacrificed at 16 weeks and tumor formation in colon was scored. Proportion of animals developing tumor(s) is shown, *N* indicates number of animals of each genotype. Statistical significance was determined by two tailed Fisher’s exact test (***p < 0.001, **p < 0.01, *p < 0.05). **c** Proportion of animals with multiple tumors in mice from **b** was scored. *N* indicates number of animals that developed at least one tumor. Statistical significance was determined by two tailed Fisher’s exact test (***p < 0.001, **p < 0.01, *p < 0.05). **d** Volume of the colon tumors developed in mice with indicated genotypes. In case of multiplicity, the largest tumor was quantified. Line indicates mean. **e** Representative sections of colon tumors from 16 weeks old *Apc*^*min*^*, Apc*^*min*^*Ppm1d*^*T/+*^ mice stained for Ki-67. Scale bars 50 and 25 μm. **f** Kaplan–Meier survival plot of mice of indicated genotypes over a period of 50 weeks. Statistical significance was determined by log rank test as follows *Apc*^*min*^ vs. *Apc*^*min*^*Ppm1d*^*T/+*^ *p* = 0.019; *Apc*^*min*^ vs. *Apc*^*min*^*Tp53*^*+/*−^ *p* = 0.008; *Apc*^*min*^*Ppm1d*^*T/+*^ vs. *Apc*^*min*^*Tp53*^*+/*−^ *p* = 0.717; *Ppm1d*^*+/+*^ vs. *Ppm1d* ^*T/+*^ *p* = 0.014). Numbers of animals per group are shown

**Fig. 5:**
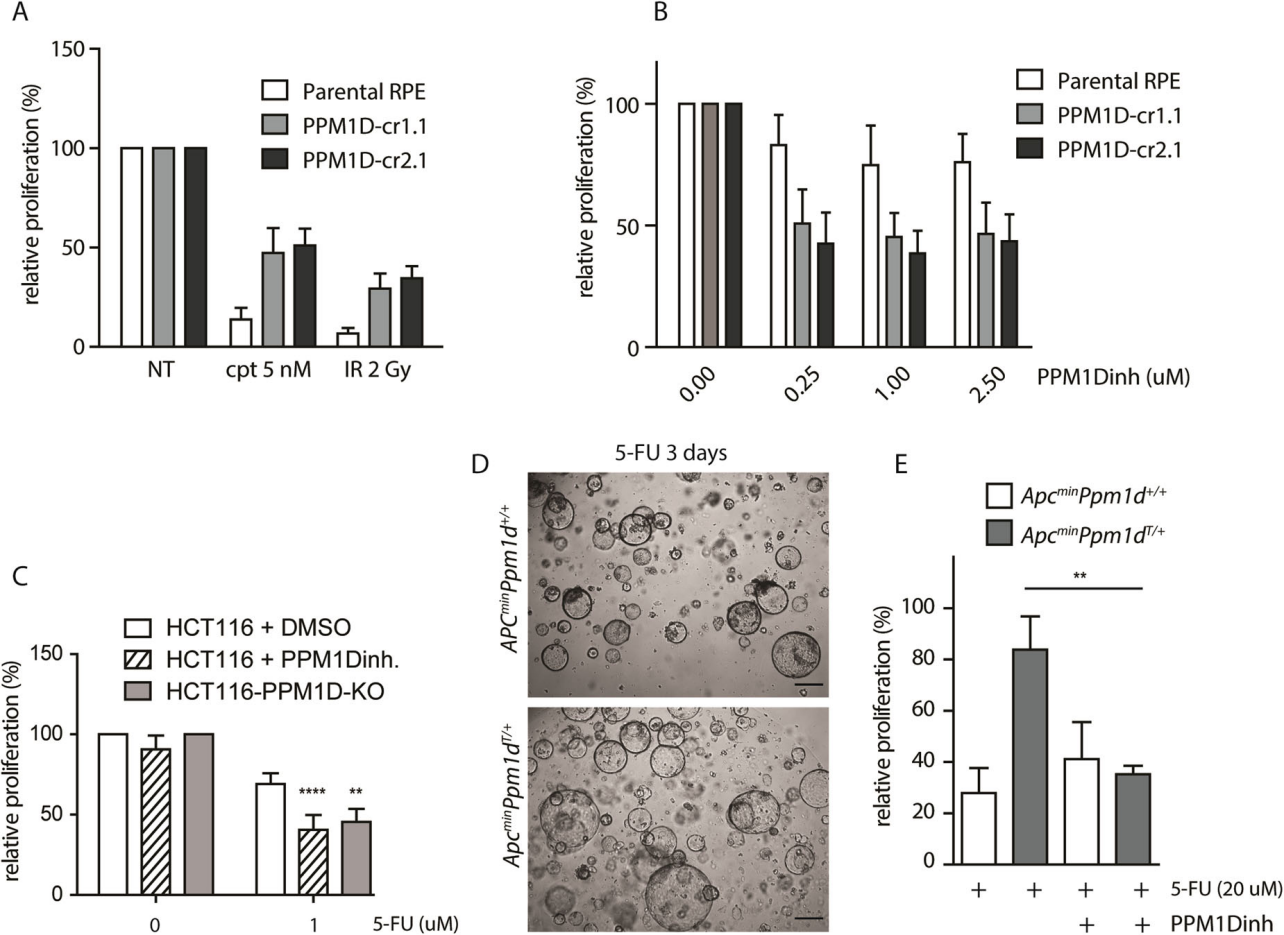
Truncation of *PPM1D* impairs sensitivity of tumor cells to chemotherapy. **a** Parental RPE, RPE-PPM1D-cr1.1 or RPE-PPM1D-cr2.1 cells were mock treated, exposed to camptothecin (5 nM) or to IR (2 Gy). Relative cell proliferation was determined after 7 days by resazurin assay (*n* = 3). **b** Parental RPE, RPE-PPM1D-cr1.1 or RPE-PPM1D-cr2.1 cells were grown in indicated doses of GSK2830371 (PPM1Dinh) for 7 days and relative cell proliferation was determined by resazurin assay. **c** Parental HCT116 cells or HCT116-PPM1D-KO cells were treated with indicated combinations of DMSO, GSK2830371 (PPM1Dinh; 0.5 µM) and 5-FU (1 µM) and relative cell proliferation was determined after 7 days by resazurin assay (*n* = 3). **d** Organoids derived from colon tumors of *Apc*^*min*^ or *Apc*^*min*^*Ppm1d*^*T/+*^ mice were grown in the presence of 5-FU for 3 days and imaged by light microscopy. Representative image is shown. Scale bar 250 µm. **e** Tumor organoids derived from *Apc*^*min*^ or *Apc*^*min*^*Ppm1d*^*T/+*^ mice (*n* > 3) were grown in the presence of 5-FU (20 µM) and GSK2830371 (PPM1Dinh; 10 µM) and survival was determined after 3 days

Loss of Ppm1d was previously shown to delay development of various solid tumors in mice including mammary and intestinal adenocarcinoma^12,49^. As the truncating *PPM1D* mutation was originally identified in HCT116 cells derived from colon adenocarcinoma^18^, we aimed to test the contribution of the truncated *Ppm1d* to tumor development in mouse intestine. Stem cell proliferation in the gastrointestinal tract is controlled by secreted glycoproteins of the Wnt family that trigger β-catenin/T-cell factor (TCF)-dependent expression of a specific set of target genes^50^. Constitutive activation of the Wnt pathway occurs in the majority of colorectal cancer (CRC) and is caused mainly by mutations in the genes encoding tumor suppressor adenomatous polyposis coli (APC) (~80% of cases), β-catenin (~4.7%), the nuclear mediator of Wnt signaling TCF4 (~4.1%) or ubiquitin ligase F-box/WD repeat-containing protein 7 (FBXW7) (~10.4%)^51^. Inactivating mutations in *APC* promote gradual development of dysplasia, adenoma, and adenocarcinoma in human cancers and corresponding mouse models^52^. Here, we crossed *Ppm1d*^*T/+*^ mice with *Apc*^*min*^ mice, an established model of gastrointestinal cancer in which the mice carry one non-functional allele of *Apc* and the second wt allele is randomly inactivated during the life span of the animals^53^. Typically, *Apc*^*min*^ mice develop multiple polyps in intestine and die approximately at 6 months of age. In contrast to humans, *Apc*^*min*^ promotes mostly small intestinal tumors, whereas tumors in the colon are less frequent^53^. As expected, multiple polyps were present in the small intestine in *Apc*^*min*^ mice and the number of polyps significantly increased in *Apc*^*min*^*Ppm1d*^*T/+*^ mice (Fig. 4a). In addition, we observed single tumors in colon in about 34% of *Apc*^*min*^ mice at 16 weeks of age (Fig. 4b, c). Strikingly, formation of the colon tumors was enhanced to 68% in *Apc*^*min*^*Ppm1d*^*T/+*^ mice and typically these mice developed multiple (usually 2–3 tumors) colon tumors at 16 weeks of age (Fig. 4b, c). Although the mean volume of the tumors in the colon was comparable in *Apc*^*min*^ and *Apc*^*min*^*Ppm1d*^*T/+*^ mice, we observed several very large tumors in a fraction of *Apc*^*min*^*Ppm1d*^*T/+*^ mice (Fig. 5d, e). The median survival of *Apc*^*min*^*Ppm1d*^*T/+*^ mice was significantly reduced when compared to *Apc*^*min*^ (28 vs. 30 weeks, log rank test, *p* = 0.019), probably reflecting a higher tumor burden in the whole intestine observed in these mice (Fig. 4f). In addition, survival of *Apc*^*min*^*Ppm1d*^*T/+*^ and *Apc*^*min*^*Tp53*^*+/−*^ was comparable (*p* = 0.717, log rank test), indicating that the impact of truncated PPM1D on promoting tumor growth was mediated by the p53 pathway. Finally, we observed significantly reduced survival of *Ppm1d*^*T/+*^ heterozygote compared to *Ppm1d*^*+/+*^ mice (*p* = 0.014, log rank test) (Fig. 4f). Within the period of 50 weeks of age we did not observe any tumor growth in the colon of *Ppm1d*^*T/+*^ mice suggesting that truncated PPM1D does not represent the tumor-initiating mutation leading to ISCs transformation. This is consistent with previous observation when overexpression of *Ppm1d* in mouse embryonic fibroblasts failed to stimulate cell growth in the soft agar but increased transformation efficiency of the RAS oncogene^15^. In summary, our data suggest that truncated *PPM1D* promotes colon tumor growth in cooperation with other driver mutations including loss of the tumor suppressor *APC*.

### Truncation of PPM1D impairs sensitivity to chemotherapy

As we observed altered response to genotoxic stress in cells carrying truncated PPM1D, we wished to address if truncated PPM1D may affect sensitivity of cells to chemotherapeutic agents. We found that RPE with truncated PPM1D proliferated in the presence of a topoisomerase inhibitor camptothecin or after exposure to the low level of IR, whereas parental RPE cells were sensitive to both treatments (Fig. 5a). Conversely, RPE with truncated PPM1D were more sensitive to GSK2830371, a specific small-molecule inhibitor of PPM1D, compared to parental RPE cells (Fig. 5b)^31,54^. These data suggested that stabilization of PPM1D can impair sensitivity of cells to DNA-damaging agents, while making them more sensitive to inhibition of PPM1D activity. To test the sensitivity in cancer cells, we used HCT116 cells that intrinsically carry PPM1D truncation, and exposed them to 5-FU, a chemotherapeutic agent commonly used in CRC treatment. We found that PPM1D inhibitor improved sensitivity of HCT116 cells to 5-FU (Fig. 5c). Similarly, genetic inactivation of PPM1D by the CRISPR/Cas9 system also sensitized HCT116 cells to 5-FU confirming the specificity of GSK2830371 (Fig. 5c). Additionally, we established organoid cultures from *Apc*^*min*^ and *Apc*^*min*^*Ppm1d*^*T/+*^ colon tumors and compared their growth in the presence 5-FU. We found that organoids derived from *Apc*^*min*^ tumors were sensitive to 5-FU, whereas *Apc*^*min*^*Ppm1d*^*T/+*^ organoids continued proliferation despite the presence of 5-FU (Fig. 5d, e). To further validate the impact of *Ppm1d*^*T*^ on resistance to 5-FU, we treated organoids with GSK2830371 and found that inhibition of PPM1D restored the sensitivity of *Apc*^*min*^*Ppm1d*^*T/+*^ organoids to 5-FU (Fig. 5e). We concluded that cancer cells carrying truncating mutations in the *PPM1D* gene are resistant to 5-FU treatment, whereas inhibition of PPM1D can restore the sensitivity to 5-FU.

### PPM1D is truncated in a fraction of colon adenocarcinomas

Finally, we wished to address potential contribution of *PPM1D* truncation to CRC development in humans. Search for the cancer cell lines harboring the truncating mutations in exon 6 of the *PPM1D* in COSMIC database (https://cancer.sanger.ac.uk/cosmic; v87) revealed that besides HCT116 cells with c.1349delT (p.L450*), similar mutations are present in SNU175 (c.1528_1529insA; p.N512Kfs*16) and CL-34 (c.1714C>T; p.R572*) cells derived from colon adenocarcinoma^55^. Next, we performed *PPM1D* mutation analysis in 364 unselected CRC samples from Norway. We found two recurrent mutations: single nucleotide deletions c.1349delT (p.L450*), and c.1535delA (p.N512Ifs*2) in five and six patients, respectively, single nucleotide duplication c.1535dupA (p.N512Kfs*16) in another patient and several individual missense variants throughout the exon 6 (Table 1, Supplementary Table S1). Interestingly, all frameshift mutations reside in two short homopolymer “hot spot” regions in exon 6, namely c.1344_1349TTTTTT and c.1529_1535AAAAAAA. Further analysis revealed that only two of 12 *PPM1D*-mutated samples also carried a mutation in *TP53* indicating that most of these tumors developed in the presence of wt p53 (Table 1). Out of the 10 *PPM1D* mutated CRC samples with wt *TP53*, all showed high level of MSI and seven carried activating *BRAF* mutations strongly suggesting that *PPM1D* mutations clustered into the consensus molecular subtype 1 (CMS1) of CRC characterized by presence of MSI and *BRAF* mutation^56,57^. Identified *PPM1D* indels resided in short homopolymer sequences, typically altered in MMR-deficient tumors^58–62^. In addition, we identified the same frameshift mutations in the hot spot region in a smaller cohort of 243 Czech colon cancer patients. Both tumors samples with truncated PPM1D carried somatic *BRAF-V600E* mutations, wt *TP53* and were classified as MSI (Table 1). Subsequently, tumors and matched non-tumor tissues were subjected to panel sequencing that revealed de novo somatic frameshift mutations in homopolymer regions of several genes including *MSH3* and *MLH3*, confirming a defect in MMR in the tumor tissue (Supplementary Table S2). Next, we screened also a cohort of 340 unselected Swedish CRC patients revealing four samples with *PPM1D* truncating mutation, which included recurrent p.L450* mutation, a deletion affecting c.1632_1636CCCCC homopolymer (c.1636delC; p.L546*), one missense mutation (c.1358C>A; p.S453*) and one insertion (c.1476dupT; p.S493*) outside the homopolymeric regions (Table 1). Finally, we searched the COSMIC database and found that *PPM1D* truncations in exon 6 harbor 89% of all *PPM1D* mutations (166/186) identified in various tumor types. The most frequent mutations occurred in samples of endometrial cancer (10/936; 1.07%), hematological tumors (46/4816; 0.96%), and CRC (29/3490; 0.83%). The distribution of exon 6 alterations further confirmed that *PPM1D* truncations in CRC, but not in other tumors, clustered into the hot spots identified in the present samples (Supplementary Fig. S3). Moreover, 23/29 (79%) mutations referred in the CRC samples in COSMIC (and 16/19; 84% in the present series) appeared in short homopolymers, in contrast with 18/64 (28%) and 2/10 (20%) of such mutations in samples from hematopoietic and endometrial tumors, respectively. Altogether, we conclude that recurrent somatic truncating *PPM1D* mutations occur dominantly in a fraction of human CRCs that remain p53-proficient and are associated with MMR pathway defects. Truncating *PPM1D* mutations in non-CRC tumors, including hematological malignancies, may arise by different mechanisms than MMR defects.

**Table 1:** Truncating *PPM1D* mutations are present in a fraction of human CRC patients and cluster into two “hot spot” codons L450 and N512 in exon 6

CRC samples (mutant/N)	*PPM1D* somatic mutation in tumor			MSI status	Other somatic mutations in tumor			Tumor localization
	cDNA (c.) change	Protein (p.) change	Mutation in homopolymer		TP53	KRAS	BRAF	
Norway (12/380)	1349delT	L450*	Yes	MSI	wt	wt	mut	Right
	1349delT	L450*	Yes	MSS	p.Y234C	wt	wt	Right
	1349delT	L450*	Yes	MSI	wt	wt	mut	Right
	1349delT	L450*	Yes	MSI	wt	wt	wt	Right
	1349delT	L450*	Yes	MSI	wt	wt	mut	Right
	1535delA	N512Ifs*2	Yes	MSI	wt	wt	mut	Right
	1535delA	N512Ifs*2	Yes	MSS	p.G245D	wt	wt	Left
	1535delA	N512Ifs*2	Yes	MSI	wt	wt	mut	Right
	1535delA	N512Ifs*2	Yes	MSI	wt	wt	mut	Right
	1535delA	N512Ifs*2	Yes	MSI	wt	wt	wt	Right
	1535delA	N512Ifs*2	Yes	MSI	wt	wt	mut	Right
	1535insA	N512Kfs*16	Yes	MSI	wt	wt	wt	Right
CZ (2/243)	1349delT	L450*	Yes	MSI	wt	wt	mut	n.a.
	1535delA	N512fs*2	Yes	MSI	wt	wt	mut	n.a.
Sweden (4/340)	1349delT	L450*	Yes	n.a.	n.a.	n.a.	n.a.	n.a.
	1358C>A	S453*	No	n.a.	n.a.	n.a.	n.a	n.a.
	1476dupT	S493*	No	n.a.	n.a.	n.a.	n.a	n.a.
	1636delC	L546*	Yes	n.a.	n.a.	n.a.	n.a	n.a.

Mutation analysis of *PPM1D*, *TP53*, *KRAS*, and *BRAF* was performed in paired non-tumor and tumor samples in CRC patients from Norway, Czech Republic and Sweden (n.a. – not available). Identified truncating somatic mutations in *PPM1D* in the tumor tissue are shown (number of identified mutants/number of tested samples). Microsatellite instability was assayed from tumorDNA (MSI, microsatellite instable; MSS, microsatellite stable)

## Discussion

By exploiting a newly generated mouse model we demonstrate that truncated Ppm1d can partially suppress the p53 pathway in ISCs in the small intestinal and colon epithelium. As a result, stem cells carrying the truncated form of Ppm1d survive in the presence of genotoxic stress whereas wt stem cells are more prone to cell death. Interestingly, *Ppm1d*^*T/+*^ and *Ppm1d*^*T/T*^ mutants showed comparable effects on suppressing p53 functions suggesting that alteration of only one *Ppm1d* allele is sufficient to induce the gain-of-function phenotype. We noted that suppression of the p53 response in *Ppm1d*^*T/+*^ mice is partial as more robust decrease of *Cdkn1a* and *PUMA* expression was observed in *Tp53* knock-out mice.

Although we did not observe increased spontaneous tumor formation in *Ppm1d*^*T/+*^ mice, we noted moderately decreased overall survival towards the end of this study (50 weeks) warranting a future follow-up of these mice. Whereas truncated PPM1D may be insufficient to fully transform ISCs and drive tumorigenesis of otherwise normal cells, in genetically sensitized background it promoted growth of colon tumors. Consistent with a previously reported suppression of the polyp formation in the small intestine in *Ppm1d* knock-out mice, we observed that truncation of Ppm1d significantly increased the number of intestinal polyps in *Apc*^*min*^*Ppm1d*^*T/+*^ mice^12^. More strikingly, we observed increased frequency of the APC-driven colon tumors in *Ppm1d*^*T/+*^ mice and their reduced survival. The phenotype is more closely resembling the tumorigenic process observed in human patients. The reason why the increased stability of PPM1D in the mouse model shifts the tumor burden from the small intestine to the colon remains unclear. We suggest the tumor-promoting role of truncated PPM1D may be related to some mutational signature that differs between small intestinal and colon tissue.

We have identified various types of somatic frameshift and/or nonsense mutations in the last exon of *PPM1D* in the colon tumors in cohorts of CRC patients from three countries and similar mutations were also found in publicly available databases. Out of these, most common are the frameshift mutations N512Ifs*2 and L450stop that are both caused by a single nucleotide deletion in the homopolymeric region in exon 6 of *PPM1D*. Tumors carrying these two mutations were typically microsatellite instable, p53-proficient, carried *BRAF* mutations and were located in the right colon. In addition, NGS of two tumors with *PPM1D* mutations revealed somatic defects in MSH genes. In summary, we conclude that the truncating *PPM1D* mutations arise primarily in MMR-deficient CRC. It will be interesting to determine the contribution of truncated *PPM1D* to tumor development using the *BRAF-V600E* mouse model.

Besides the two recurrent frameshift mutations, we identified several individual nonsense mutations outside the hotspots. Similar nonsense *PPM1D* mutations were recently found in a fraction of HSCs and their frequency typically increases with advancing age^22^. Clonal hematopoiesis may represent a serious threat when resistant cell clones are selected during chemotherapy and the clones promote development of secondary malignancies including therapy-related AML and myelodysplastic syndrome^24^. With new technological advances in sequencing of individual stem cells, it is now becoming clear that similar age-related clonal differences in the stem cell populations may occur also in other tissues including the small intestine, colon, and liver^63^. We hypothesize that some of the *PPM1D* truncating mutations occur randomly in a fraction of colon stem cells, providing a selective advantage compared to healthy stem cells and potentially contributing to cancer development.

Using cell competition assays, hematopoietic cells carrying truncated PPM1D were recently shown to outcompete wt cells when transplanted into lethally irradiated mice treated with cytarabine, cisplatin, or etoposide^22,24^. Here, we tested the sensitivity of organoid cultures derived from *Apc*^*min*^ or *Apc*^*min*^*Ppm1d*^*T/+*^ colon tumors to 5-FU, used as a standard chemotherapy for CRC. We found that *Apc*^*min*^*Ppm1d*^*T/+*^ organoids were less sensitive to 5-FU compared to *Apc*^*min*^ organoids. Importantly, inhibition of PPM1D by a small molecule inhibitor increased sensitivity of organoids carrying truncated *PPM1D* to 5-FU, likely reflecting increased activity of p53, which is an important factor determining the sensitivity to 5-FU^64^. Allosteric inhibitor GSK2830371 potently suppresses the PPM1D activity; however, its low stability prevented us from testing its efficiency directly in mice. Recently it has been suggested that combining chemotherapy with inhibition of PPM1D could prevent development of secondary therapy-related tumors^22^. Based on our results we propose that inhibition of PPM1D may represent a promising strategy for improving efficacy of 5-FU in colon cancer treatment, especially in patients with MMR-deficient tumors located in caecum and ascending colon.

## Supplementary information

Supplementary figure 1

Supplementary figure 2

Supplementary figure 3

Supplementary table 1

Supplementary table 2

Contribution form
