# Supplementary figures and images for: Truncated PPM1D impairs stem cell response to genotoxic stress and promotes growth of APC-deficient tumors in the mouse colon
Source: Cell Death Dis. 2019 Oct 28;10(11):818. doi: 10.1038/s41419-019-2057-4 (PMC6817818; doi:10.1038/s41419-019-2057-4)

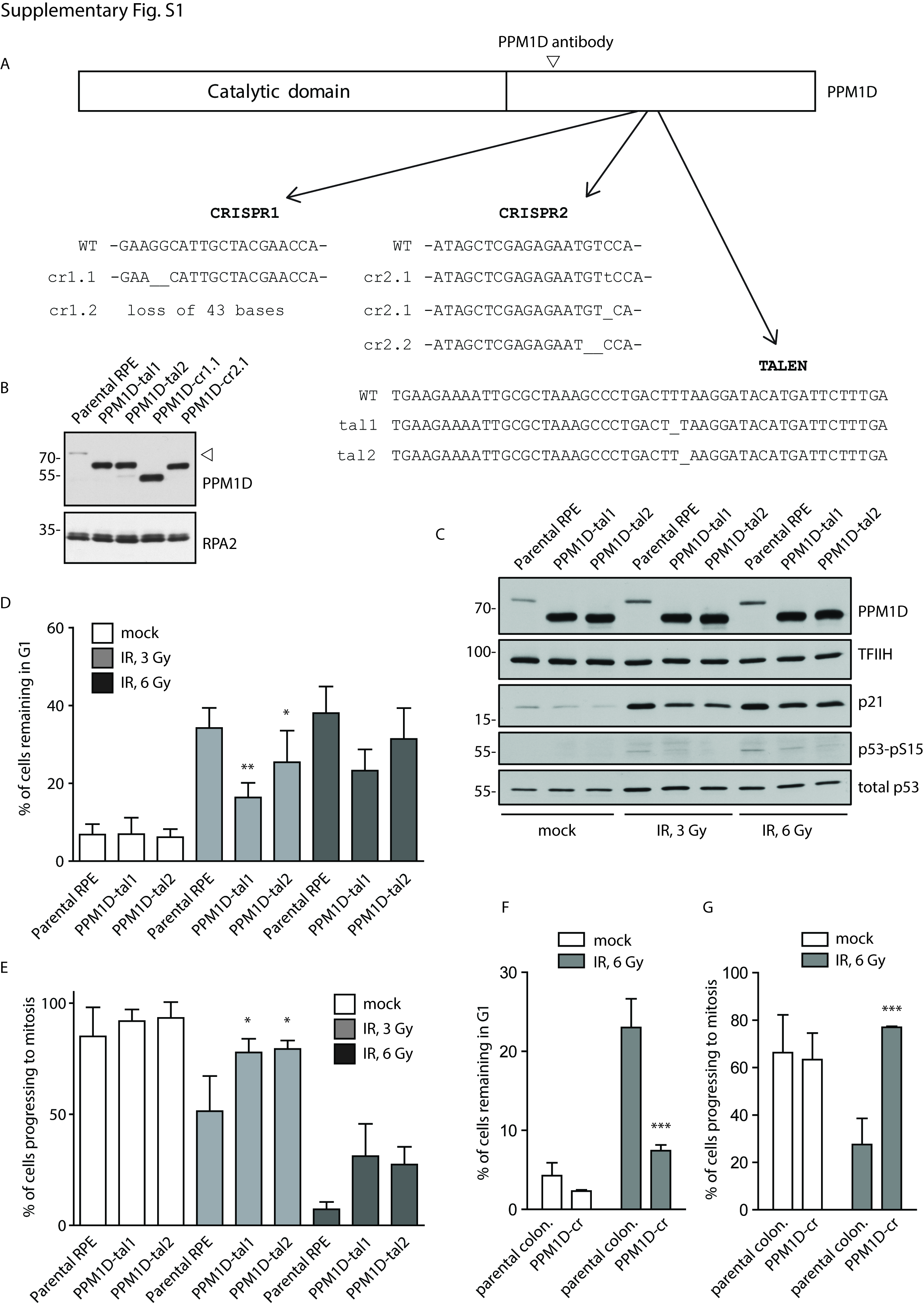

Supplement: Supplementary file 1 — Supplementary figure 1 [file 41419_2019_2057_MOESM1_ESM.tif]

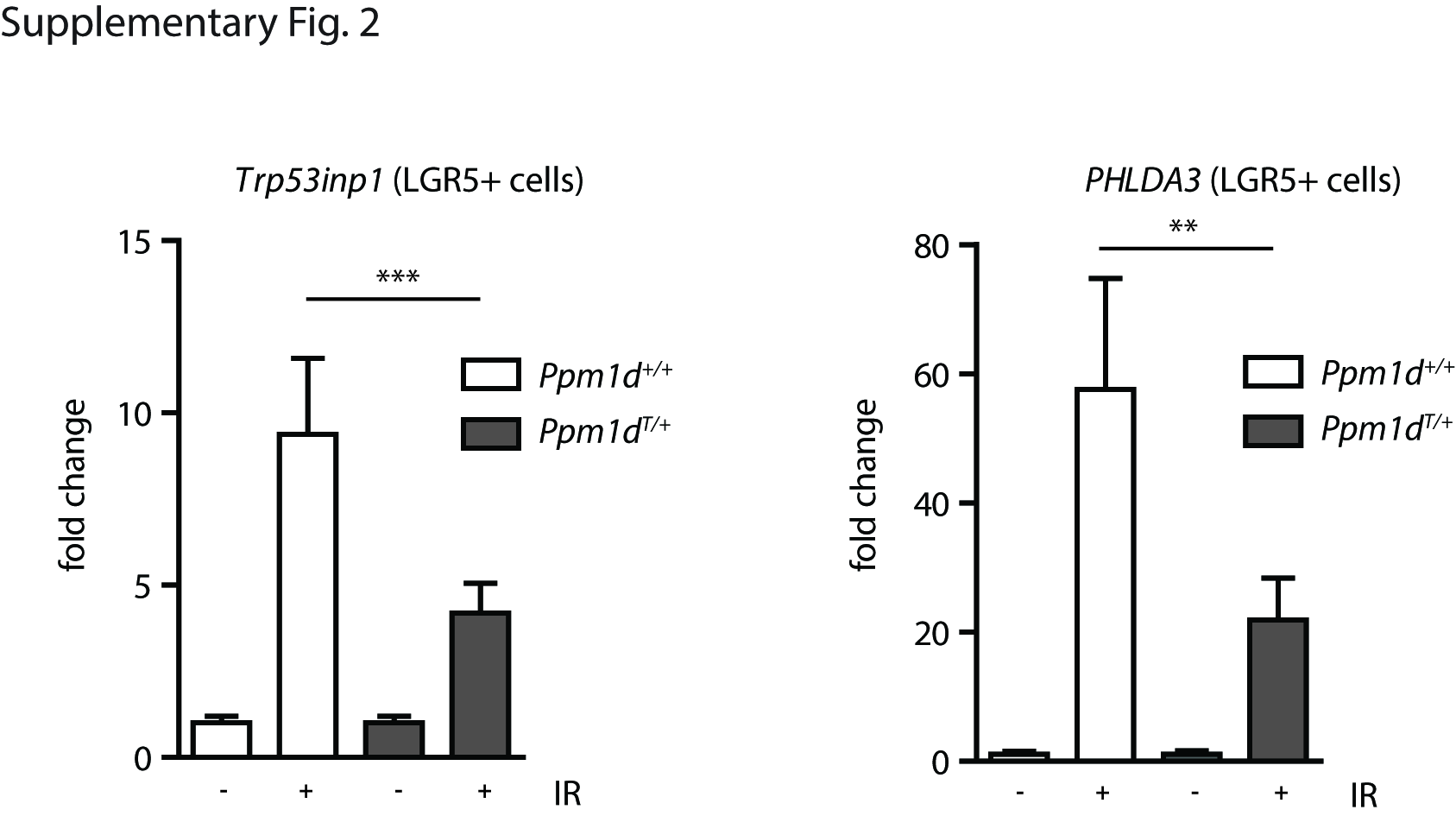

Supplement: Supplementary file 2 — Supplementary figure 2 [file 41419_2019_2057_MOESM2_ESM.tif]

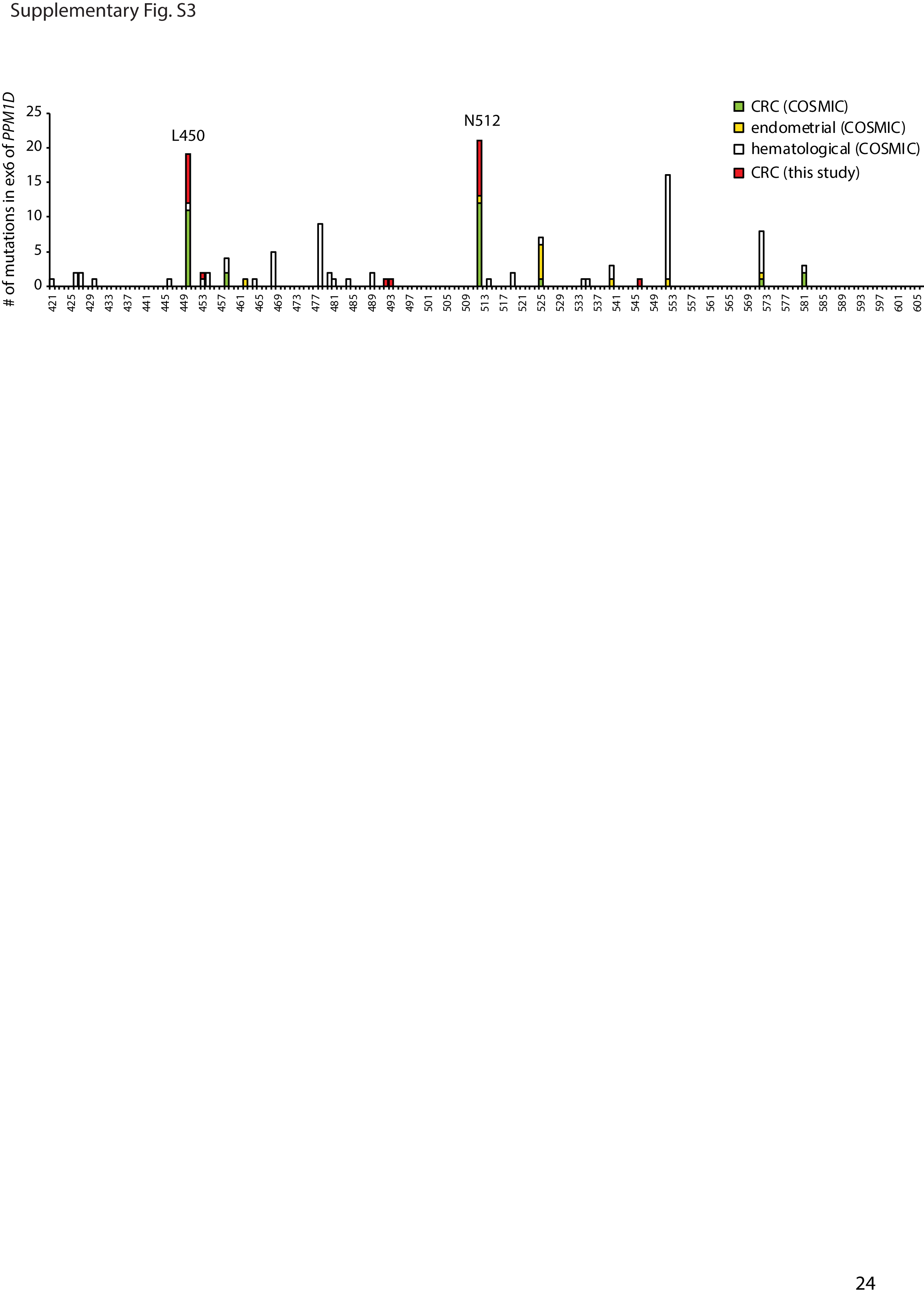

Supplement: Supplementary file 3 — Supplementary figure 3 [file 41419_2019_2057_MOESM3_ESM.tif]
