# Supplementary material for: Truncated PPM1D impairs stem cell response to genotoxic stress and promotes growth of APC-deficient tumors in the mouse colon
Source: Cell Death Dis. 2019 Oct 28;10(11):818. doi: 10.1038/s41419-019-2057-4 (PMC6817818; doi:10.1038/s41419-019-2057-4)
Supplement: Supplementary file 6 — Contribution form [file 41419_2019_2057_MOESM6_ESM.pdf]

DECLARATION OF CONTRIBUTIONS TO ARTICLE

ADMC

Manuscript Number: CDDIS-19-2385R

Journal Name: *Cell Death & Disease* (the 'Journal')

Proposed Title of the Contribution:

Truncated PPM1D impairs stem cell response to genotoxic stress and promotes growth of APC-deficient tumors in the mouse colon (the 'Contribution')

Author(s):

Monika Burocziova, Kamila Burdova, Andra S. Martinikova, Petr Kasparek, Petra Kleiblova, Aske S. Danielsen, Marianna Borecka, Gabriela Jeníkova, Lucie Janečková, Jozef Pavel, Petra Zemánkova, Michaela Schneiderova, Lucie Schwarzova, Ivana Ticha, Xiao-Feng Sun, Katerina Jiraskova, Vaclav Liska, Ludmila Vodickova, Pavel Vodicka, Radislav Sedlacek, Zdenek Kleibl, Ragenhild A. Lothe, Vladimír Korinek, Libor Macurek (the 'Authors')

For all *CDDis* articles, each person named as an author in the published version must be able to show he or she has contributed substantially to the article.

Authorship credit should be based on 1) substantial contributions to conception and design, acquisition of data, or analysis and interpretation of data; 2) drafting the article or revising it critically for important intellectual content; and 3) final approval of the version to be published. Authors should meet conditions 1, 2 and 3.

Any person who cannot be shown to have made a substantial contribution to the article cannot be listed as an author in the final version. The name of any person who is deemed to have made a minor contribution can, however, appear in the Acknowledgments section of the article.

Please complete the table below to indicate the contributions of all named authors to the manuscript.

Author Full Name:	Specification of Contribution to the Manuscript:
Monika Burocziova	acquisition of data, analysis, interpretation of data
Kamila Burdova	acquisition of data, analysis, interpretation of data
Andra S. Martinikova	acquisition of data, analysis, interpretation of data
Petr Kasparek	acquisition of data, analysis, interpretation of data
Petra Kleiblova	acquisition of data, analysis,
Aske S. Danielsen	acquisition of data, analysis,
Marianna Borecka	acquisition of data, analysis,
Gabriela Jeníkova	acquisition of data, analysis,
Lucie Janečková	acquisition of data, analysis,
Jozef Pavel	acquisition of data, analysis,
Petra Zemánkova	acquisition of data, analysis,
Michaela Schneiderova	acquisition of data,
Lucie Schwarzova	acquisition of data,

DECLARATION OF CONTRIBUTIONS TO ARTICLE

ADMC

Manuscript Number: CDDIS-19-2385R

Journal Name: *Cell Death & Disease* (the 'Journal')

Proposed Title of the Contribution:

Truncated PPM1D impairs stem cell response to genotoxic stress and promotes growth of APC-deficient tumors in the mouse colon (the 'Contribution)

Author(s):

Monika Burocziova, Kamila Burdova, Andra S. Martinikova, Petr Kasparek, Petra Kleiblova, Aske S. Danielsen, Marianna Borecka, Gabriela Jeníkova, Lucie Janečková, Jozef Pavel, Petra Zemánkova, Michaela Schneiderova, Lucie Schwarzova, Ivana Ticha, Xiao-Feng Sun, Katerina Jiraskova, Vaclav Liska, Ludmila Vodickova, Pavel Vodicka, Radislav Sedlacek, Zdenek Kleibl, Ragenhild A. Lothe, Vladimír Korinek, Libor Macurek (the 'Authors')

For all *CDDis* articles, each person named as an author in the published version must be able to show he or she has contributed substantially to the article.

Authorship credit should be based on 1) substantial contributions to conception and design, acquisition of data, or analysis and interpretation of data; 2) drafting the article or revising it critically for important intellectual content; and 3) final approval of the version to be published. Authors should meet conditions 1, 2 and 3.

Any person who cannot be shown to have made a substantial contribution to the article cannot be listed as an author in the final version. The name of any person who is deemed to have made a minor contribution can, however, appear in the Acknowledgments section of the article.

Please complete the table below to indicate the contributions of all named authors to the manuscript.

Author Full Name:	Specification of Contribution to the Manuscript:
Ivana Ticha	acquisition of data, analysis,
Xiao-Feng Sun	acquisition of data, analysis, interpretation of data
Katerina Jiraskova	acquisition of data, analysis,
Vaclav Liska	acquisition of data,
Ludmila Vodickova	acquisition of data, analysis, interpretation of data
Pavel Vodicka	acquisition of data, analysis, interpretation of data, revising manuscript
Radislav Sedlacek	conception and design, grant support
Zdenek Kleibl	acquisition of data, analysis, revising manuscript
Ragenhild A. Lothe	conception and design, grant support, revising manuscript
Vladimír Korinek	conception and design, revising manuscript
Libor Macurek	acquisition of data, analysis, interpretation of data, conception and design, grant support, writing the manuscript
	All authors approved the final version of the manuscript

Please complete the table below to indicate the contributions of all named authors to the figures.

Figure 1:

Kamila Burdova, Andra S. Martinikova

Figure 2:

Monika Burocziova

Figure 3:

Monika Burocziova, Andra S. Martinikova, Petr Kasparek, Gabriela Jeníkova,Lucie Janečková, Jozef Pavel

Figure 4:

Monika Burocziova, Andra S. Martinikova

Figure 5:

Monika Burocziova, Lucie Janečková

Figure 6:

Table 1 and supplementary fig 3 and tables 1 and 2: Petra Kleiblova, Aske S. Danielsen, Marianna Borecka, Petra Zemánkova, Michaela Schneiderova, Lucie Schwarzova, Ivana Ticha, Xiao-Feng Sun, Katerina Jiraskova, Vaclav Liska, Ludmila Vodickova, Pavel Vodicka, Zdenek Kleibl

Signed for and on behalf of the Author(s):	Print Name:	Date:
[signature]	Libor Macurek	October 4, 2019
